# A multi-representation approach to the contextual interference effect: effects of sequence length and practice

**DOI:** 10.1007/s00426-021-01543-0

**Published:** 2021-06-16

**Authors:** Willem B. Verwey, David L. Wright, Maarten A. Immink

**Affiliations:** 1 Department of Learning, Data-Analytics and Technology Cognition, Data and Education Section, Faculty of Behavioural, Management and Social Sciences, University of Twente, PO Box 217, 7500 AE Enschede, The Netherlands; 2 Department of Kinesiology, Texas A&M University, College Station, TX USA; 3 Sport, Health, Activity, Performance and Exercise Research Centre Flinders University, Adelaide, Australia

## Abstract

The present study investigated the long-term benefit of Random-Practice (RP) over Blocked-Practice (BP) within the contextual interference (CI) effect for motor learning. We addressed the extent to which motor sequence length and practice amount factors moderate the CI effect given that previous reports, often in applied research, have reported no long-term advantage from RP. Based on predictions arising from the Cognitive framework of Sequential Motor Behavior (C-SMB) and using the Discrete Sequence Production (DSP) task, two experiments were conducted to compare limited and extended practice amounts of 4- and 7-key sequences under RP and BP schedules. Twenty-four-hour delayed retention performance confirmed the C-SMB prediction that the CI-effect occurs only with short sequences that receive little practice. The benefit of RP with limited practice was associated with overnight motor memory consolidation. Further testing with single-stimulus as well as novel and unstructured (i.e., random) sequences indicated that limited practice under RP schedules enhances both reaction and chunking modes of sequence execution with the latter mode benefitting from the development of implicit and explicit forms of sequence representation. In the case of 7-key sequences, extended practice with RP and BP schedules provided for equivalent development of sequence representations. Higher explicit awareness of sequence structures was associated with faster completion of practiced but also of novel and unstructured sequences.

## Introduction

### The contextual interference effect

A recurring question in skills research concerns how new motor skills can be best practiced. The optimal practice regime combines limited practice time with rapid improvement, high long-term retention, and robustness in adapting to novel skill variations. To achieve this, guidelines often imply that teachers, instructors, coaches, and clinicians should strive for high *contextual interference* (CI). CI is high in a *Random-Practice* (RP) regime in which several motor sequences are constantly alternated during practice. CI is low when individual motor sequences are practiced extensively in separate blocks in a *Blocked-Practice* (BP) regime. The benefit of high over low CI training for long term retention, the so-called *CI-effect*, has been demonstrated in many studies over the past forty years (Brady, 2004; Magill & Hall, 1990; Shea & Morgan, 1979; for recent reviews, see Farrow & Buszard, 2017; Immink, Verwey & Wright, 2020; Wright et al., 2016). Often this long-term learning advantage of RP over BP is accompanied by reduced performance and slower improvement during RP than BP.

The *elaborative-processing* hypothesis posits that RP forces learners into more elaborate processes than BP, like inter-task comparisons and embellishment of task-relevant information (Shea & Morgan, 1979; Shea & Zimny, 1983). Learners, therefore, appreciate better the distinctiveness of the different tasks and are better able to retrieve alternative representations. Instead, the *forgetting-reconstruction* hypothesis assumes that repeated construction of the motor plan in short-term memory strengthens the relevant memory representations (Lee & Magill, 1983, 1985). During RP this preparation occurs at each trial while during BP it occurs basically only at the start of a block after which the motor plan remains available in short-term memory (cf. Haith & Krakauer, 2018; Rosenbaum et al., 1986). There is indeed ample evidence that RP involves greater attention and response planning demands than BP (e.g., Immink & Wright, 1998; Li & Wright, 2000; Sheahan et al., 2016; for a review, see Wright et al., 2016). However, these greater demands may be instrumental for improved learning instead of supporting a particular hypothesis (Pauwels, Swinnen, & Beets, 2014; Young et al., 1993). In fact, increased cognitive activity may reflect a *desirable difficulty* (Bjork, 1994; Christina & Bjork, 1991) that prompts the offline knowledge consolidation that is now generally assumed to be responsible for the learning advantage associated with high CI (Kantak et al., 2010; Kim & Wright, 2020; Lin et al., 2011).

Despite demonstrations that the CI-effect holds in a variety of situations (Lee, 2012) and with various populations (e.g., people with Down’s syndrome, Edwards et al., 1986; and with a mild mental handicap, Porretta & O'brien, 1991), the merit of RP over BP for learning does not seem to extend to all motor learning situations. A review of applied studies on the effect of CI reported a beneficial effect of high CI in just 58% of the 27 reviewed studies (Barreiros et al., 2007). The authors established that the CI-effect occurs primarily in tasks of a serial nature and not in propulsive tasks like throwing. It has further been argued that the CI-effect is observed especially in simple and modestly complex motor skills in closed, predictable environments (Brady, 2008; Farrow & Buszard, 2017; Wulf & Shea, 2002). As these are the situations that allow for extended motor plan preparation, one wonders whether perhaps motor learning is fostered especially by repeated motor preparation rather than by motor execution (Immink & Wright, 1998; Li & Wright, 2000). Finally, there are indications that the CI-effect occurs only with modest numbers of practice trials (Perez, Meira Jr, & Tani, 2005). Indeed, CI studies often involve less than 50 practice trials. For example, studies by Cross et al. (2007), Immink and Wright (1998) and Shea and Morgan (1979) involved only 18 practice trials per movement sequence. So, while there is substantial evidence for the CI-effect, its scope may be limited to motor sequences of limited length that have been practiced only briefly.

### Discrete motor sequences and C-SMB

The purpose of the present two experiments was to explore potential limits of the CI-effect with the Discrete Sequence Production (DSP) task (Verwey, 1999, 2001; for a review see Abrahamse et al., 2013). This task typically involves two fixed series of 6 or 7 successive key presses. Learning these discrete motor sequences has previously been found to also benefit from high CI (Cross et al., 2007; Immink & Wright, 1998; Kim et al., 2018). CI research with the DSP task has the advantage that predictions can be tested using the *Cognitive framework for Sequential Motor Behavior* (C-SMB, Verwey et al., 2015, which extended the Dual Processor Model, Abrahamse et al., 2013). This framework posits that the execution of familiar motor sequences can be controlled by a slow and flexible central processor and a fast and inflexible motor processor. This assumption receives support from fMRI studies showing that sequence learning engages various different neural networks the activity of which depends on the skill level (Verwey et al., 2019).

The central processor (also denoted 'cognitive processor' in earlier articles, e.g., Abrahamse et al., 2013) is assumed to initially trigger individual responses using key-specific stimuli and stimulus–response mappings in a *reaction mode*. After tens of practice trials, *central-symbolic sequence representations* develop that code the order of the movements spatially and/or verbally.^1^ The verbal, and to a lesser extent the spatial, representations are accessible to consciousness and therefore largely explicit (cf. Keele et al., 2003). Selecting individual movements from these central-symbolic representations by the central processor during sequence execution is a cognitively demanding and relatively slow process (Verwey & Wright, 2014). Recent research suggests that central-symbolic representations develop as a function of how often they have been prepared or imagined, and that physical execution may not be needed to develop these representations (Sheahan et al., 2016; Sobierajewicz et al., 2017).

With hundreds of practice trials, motor sequences are learned also at the motor level of processing. This is indicated by the execution of motor sequences becoming fast, relying less on cognitive processes, and involving effector-specific components and coarticulation of individual effectors. C-SMB attributes this to the development of *motor chunks* that are selected by the central processor after which they are executed by the motor processor. Motor chunks would include only a limited number of responses (Abrahamse et al., 2013; Verwey, 2001), but multiple studies have shown that the indications for segmentation of longer sequences vanish with extended practice (Acuna et al., 2014; Ramkumar et al., 2016; Wymbs et al., 2012). This suggests that sequence learning at the motor level is based on response-response associations that do not impose a length restriction (Lindsey & Logan, 2019; Logan, 2020). This explains findings that the execution rate of individual responses of a well-known motor sequence increase with sequence position (Lehiste, 1970; MacKay, 1982, 1990). In retrospect, several DSP task studies show support for this view in that after extended practice execution rate increased and also became more independent from key-specific stimuli at the end of the sequence (Brown & Carr, 1989; Verwey, 1994, 2021; Verwey, Wright, & van der Lubbe, 2020). To emphasize that at the motor level sequence representations are not limited by short-term memory capacity, below we use the term *sequential motor representation* instead of motor chunk.^2^

The concurrent availability of central-symbolic and sequential motor representations that are used by different processors implies that executing familiar motor sequences involves a race between these two processors. That is, after having initiated a sequential motor representation that is then carried out by the motor processor, the central processor can in parallel still trigger individual movements using key-specific stimuli or central-symbolic representations.

### Contextual interference and discrete sequence learning

The suggestion that the retention benefit of high CI reduces as movement sequences get more complex (Brady, 2008; Farrow & Buszard, 2017; Wulf & Shea, 2002) can be accounted for by the adjusted version of C-SMB. The limitation of short-term memory to about 4 items (Cowan, 2000) prevents preparation and execution of longer sequences as a whole so that these sequences are carried out as a succession of a few short segments (Verwey, 1996; Verwey et al., 2009). In line with the forgetting-reconstruction hypothesis (Lee & Magill, 1983, 1985), we assume that repeated preparation of response series in short-term memory induces the development of central-symbolic representations. In the case of sequences of more than about 4 responses these representations need to be concatenated to produce the entire motor sequence. Even though the central processor can already select each oncoming segment during the execution of the preceding segment (Garcia-Colera & Semjen, 1987; van Donkelaar & Franks, 1991; Verwey, 1995, 2001), concatenation is usually still indicated by a relatively slow response (Abrahamse et al., 2013; Acuna et al., 2014; Verwey, 1996, 2001; Verwey & Dronkers, 2019; Verwey & Eikelboom, 2003; Wymbs et al., 2012). That short-term memory is indeed involved is corroborated by findings that individual differences in short-term visuospatial short-term memory capacity predict the temporal structure of movement sequences (Bo & Seidler, 2009; Seidler et al., 2012). Importantly, the need to prepare successive segments in sequences of over about 4 responses predicts that CI increases during BP and that this may reduce or even nullify the RP benefit for retention with these longer sequences. So, the notion that central-symbolic representations develop quickly as the result of repeated preparation in short-term memory predicts that with moderate practice sequencing skill develops more rapidly during RP than BP for sequences up to about 4 responses, but this benefit of high CI disappears when sequences get longer.

The C-SMB framework predicts that after extended practice, when sequential motor representations become dominant, sequence performance is independent of whether previous practice involved RP or BP because these motor representations develop primarily from the number of times the sequence is executed. High volumes of sequence execution allow for representation of the entire sequence.

### Additional skills

The above reasoning concerns the development of representations of motor sequences. However, RP appears to also benefit the development of a *general sequence execution skill*, in that novel sequences benefit from RP too. This has been observed with the original barrier knock-down task (Shea & Morgan, 1979), and emerged also with the serial reaction time task (Kim et al., 2016; Müssgens & Ullén, 2015) and with a paced, single-finger, discrete keying sequence task (Hodges et al., 2014). The frequent use of response-specific stimuli while practicing motor sequences suggests that improved execution of novel sequences after RP may also be caused by an improved skill to respond to key-specific stimuli, a general *reaction skill* (Hommel et al., 2001; Logan, 1988; Pashler & Baylis, 1991). Hence, practicing DSP sequences might not only strengthen sequence-specific representations but also improve the more general skills to execute motor sequences and react to stimuli.

### The present study

This study addressed two main issues. First, we tested with the DSP task the prediction that the well-known retention advantage of RP over BP is limited to short keying sequences that are briefly practiced. We also explored whether this advantage is indeed associated with overnight consolidation (Diekelmann & Born, 2010; Kim & Wright, 2020; McGaugh, 2000; Wright et al., 2010). Second, we investigated the nature of the CI effect in that RP may benefit not only the development of sequence-specific representations, but also general sequencing and reaction skills. We pursued also the possibility that the performance advantage of RP over BP may involve an explicit sequence knowledge component.

These issues were investigated with two experiments that started off with practicing three discrete keying sequences on Day 1 followed by a test phase on Day 2. Both experiments involved a *Limited-Practice group* practicing each sequence on Day 1 for 24 trials (each trial involving an entire sequence), and an *Extended-Practice group* practicing on Day 1 each sequence for 504 trials. Experiment 1 involved practicing three 4-key sequences, each of which was expected to be represented by a single central-symbolic representation that allows full preparation in short-term memory. Experiment 2 was identical to Experiment 1 except that the three sequences included 7 key presses that most likely cannot be prepared as a whole so that after moderate practice each one would include two successive central-symbolic representations.

The two experiments involved the same four test conditions on Day 2. This test phase started off with the *Retention condition* to test the prediction that RP participants show better retention than BP participants after limited practice of the 4-key sequences while this CI effect would not occur with extended practice of 4-key sequences and with the 7-key sequences. Overnight consolidation in the various practice conditions was evaluated by comparing performance during retention on Day 2 with performance at the end of Day 1.

To unveil the processes that benefit from RP the participants served in three more test conditions. The Retention condition was followed by two conditions in a counterbalanced order. The *Novel* condition was used to replicate transfer of the RP benefit to novel sequences (Hodges et al., 2014; Kim et al., 2016; Müssgens & Ullén, 2015; Shea & Morgan, 1979). The *Unstructured* condition specifically tested whether reaction skill would benefit from earlier RP as this condition consisted of short series of randomly selected stimuli so that participants were forced to continue responding to each key-specific stimulus.

The last test condition entailed the *Single-stimulus* condition in which participants performed the practiced sequences in response to just the first stimulus. This condition was used to examine whether the expected learning benefit of RP over BP can be attributed to improved sequence representations and general sequence execution skill when reactions are not possible. Here performance was expected to be slower and therefore be based more on explicit sequence knowledge.

All test conditions included a blocked protocol (like during BP) to make sure that the expected better retention on Day 2 after RP on Day 1 cannot be attributed to the use of the same testing protocol as used during RP (e.g., Wright et al., 2004). Such a blocked protocol does imply that the focus of this study is on the effects of CI on the skill to execute sequences rather than on the selection and preparation of these sequences.

At the end of Days 1 and 2 participants performed an awareness task to assess explicit knowledge of the three practiced sequences. We used this to examine whether the expected beneficial effect of greater CI in the DSP sequences is perhaps associated with the development of explicit sequence knowledge. Table 1 summarizes the processing skills and knowledge types assessed in the four test conditions.

**Table 1:** Types of knowledge and skill assumed to be required in the four test conditions on Day 2 of Experiments 1 and 2

Learning benefit → test condition ↓	General reaction skill	General sequence execution skill	(implicit and explicit) sequence-specific knowledge
Retention	√	√	√
Unstructured	√		
Novel	√	√	
Single-stimulus		√	√

In the Limited-Practice group, sequence-specific knowledge would involve central-symbolic knowledge and not sequential motor representations

## Experiment 1

### Method

#### Participants

Forty-eight students from the University of Twente and Saxion University of Applied Sciences participated in exchange for either course credits or financial reward. We used 48 participants as that implies 12 participants per practice and CI-Group. This number is quite typical for DSP studies, and it also allows full counterbalancing of the 4 sequences we used in this experiment (3 practice and 1 novel sequence). All subjects indicated to be right-handed, non-smokers, and not having used alcohol 24 h prior to the experiment to prevent substance-induced inattention. They were between 20 and 29 years old and had a mean age of 22, and 24 were male. The participants were randomly assigned to the 4 participants groups (Limited/Extended-Practice x Low/High CI-Group) in a chronological order. None of the participants was familiar with the experimental task. This study was approved by the ethics committee of the Faculty of Behavioural, Management, and Social Sciences at the University of Twente.

#### Apparatus

The experiment ran on a Dell Optiplex 9010 PC under Windows 7. We used E-Prime 2.0 for both the DSP and the awareness tasks. Unnecessary Windows services were shut down to improve the measurement of response times (RTs). The stimuli were presented on a 22-inch LG FLATRON E2210PM-BN LCD screen with 1280 × 1024 pixel resolution, using a Logitech Deluxe 250 USB keyboard. Viewing distance was approximately 50–60 cm. Progress of the experiment was monitored via a GoPro observation camera.

#### DSP task

Four empty, black-border, equally distanced placeholders, consisting of 2 × 2 cm squares, were displayed in the middle of a white screen in a horizontal row that represented the placement of the C, V, B, and N keys on the PC keyboard. The participants placed the four fingers of their right hand on the spatially compatible keys. As soon as one of the squares was filled with green, the participants pressed the corresponding key. Immediately after pressing the correct key, the corresponding stimulus square changed back to white and the following square turned green until the sequence had been completed. The screen then turned white for 1000 ms. Subsequently, the four squares appeared on the screen again and after 1000 ms the first stimulus of the next sequence appeared. In case an incorrect key was pressed, the sequence was broken off, an error message appeared for a duration of 2000 ms, which was followed by 1000 ms of a white screen and another 1000 ms with the four squares present on the screen until the beginning of the next sequence. This particular sequence was not repeated later. If the participants pressed the key before the appearance of the first stimulus, a “Too early” warning appeared, immediately after which the sequence was repeated. In total, the task included four different sequences: CBNV, NCBC, BCVN, and VNCB. Three of the sequences were used for practice, the fourth one constituted the novel (i.e., the unfamiliar) sequence in the test phase. We counterbalanced which of these sequences would be the novel sequence. Stimuli, responses, and the time between each stimulus and responses are designated by S, R, and T followed by an index showing their position in the sequence (yielding in Experiment 1 S_1_–S_4_, R_1_–R_4_, and T_1_–T_4_, respectively). A trial involves execution of an entire sequence.

#### Awareness task

The awareness task included two awareness tests that were administered in a counterbalanced order to assess explicit spatial and verbal sequence knowledge (Verwey & Dronkers, 2019; Verwey et al., 2020). In the *Spatial awareness test*, the four placeholders were displayed horizontally, just like the placeholders in the DSP task. Participants were instructed to click with a mouse each of these four placeholders in the order of each of the three practiced sequences. No performance feedback was given after completing this task. The assumption was that using a different response method would reduce the possibility to use implicit sequence knowledge, and performance relies more heavily on the global availability of sequence knowledge (Baars, 1988; Rünger & Frensch, 2010). In the *Verbal awareness test*, the letters of the four keys participants had been pressing during practice were displayed in placeholders located in a rhombus shape. Participants were to indicate the order of the letters of the keys they had been pressing for each of the three sequences by clicking the appropriate placeholder. This test required verbal knowledge of the key order.

Participants had only one chance during the awareness task to indicate the response order of their two sequences in the spatial and in the verbal awareness test—four sequences reproductions in total. To prevent the use of key locations, participants could not see the keyboard during these tests. They were told that not knowing their sequences was not a problem. We analyzed both the number of entirely correct sequences and the number of correct mouse clicks at each sequence position.

#### Procedure

On Day 1, participants practiced under a BP or RP regime in either a Limited-Practice group or an Extended-Practice group (Table 2). The Limited-Practice group practiced three sequences for 24 trials each. The Extended-Practice group practiced the same three DSP sequences in six consecutive blocks, with a total of 504 practice trials per sequence. For the Limited-Practice group Blocked-Practice (BP) was induced by having participants practice each of the three sequences in three successive, 24-trial blocks. The Extended-Practice group practiced each sequence in two successive 252-trial blocks, thus yielding 6 practice blocks. Random-Practice (RP) was induced by the software preparing for each block a set of 24 (Limited-Practice group) or 252 trials (Extended-Practice group). These sets consisted of equal numbers of the three sequences, hence three sets of either 8 or 84 trials of each sequence. On each trial, the software randomly picked from the prepared set a sequence, without replacement. This made sure that the order was randomized while at the end of each block the same numbers of trials had been executed with each sequence.

**Table 2:** The procedure used with the limited- and extended-practice groups

	Limited-Practice group	Extended-Practice group
Day 1	**Practice phase** 3 24-trial blocks (*n* = 12 BP + 12 RP participants; total 24 trials/sequence)	**Practice phase** 6 252-trial blocks (*n* = 12 BP + 12 RP participants; total 504 trials/sequence)
	Awareness Task 1 (Spatial & Verbal awareness test)	
Day 2	**Test phase** (*n* = 48 participants) Part 1: Retention condition: 3 blocked subblocks (12 trials of each sequence) Part 2: 2 blocks, counterbalanced order of: –Unstructured condition: 3 subblocks (12 trials) –Novel condition: 3 blocked subblocks novel sequence (12 trials) Part 3: Single-stimulus condition: 3 blocked subblocks (12 trials)	
	Awareness Task 2 (Spatial & Verbal awareness test)	

Each participant practiced three 4-key sequences on Day 1. Day 1 and Day 2 were always consecutive

Halfway through each block, that is, after 12 or 126 trials, performance feedback in terms of average response time and error rate were displayed. The first subblock was then followed by a 40-s pause which involved counting down from 40 to 0. At the end of the second subblock (i.e., the end of the block) performance feedback was followed by a 240-s pause that also involved a second counter. At the end of the 240-s break, the experimenter entered the room and started the next block.

The test phase on Day 2 was identical for all four groups. It consisted of three parts. Part 1 tested retention with the DSP task and contained three subblocks, each including one of the three practiced sequences. In Part 2, participants performed in two blocks, each consisting of 3 subblocks. One block involved the Novel condition. It included the fourth of our set of four fixed sequences, of which the other three were used for practice. The other block of Part 2 involved the Unstructured condition. It consisted of series of 4 successive reactions to stimuli that were randomly selected before being displayed (no repetitions). So, while participants could learn the novel sequence in the Novel condition, they could merely react to the unpredictable stimuli in the Unstructured condition. Finally, Part 3 involved the Single-stimulus condition. We used it to explore whether participants were able to reproduce their 3 practiced sequences in response to just the first stimulus. At the end of Day 1 (practice phase) and of Day 2 (test phase) participants performed the awareness task in which they clicked with the computer mouse the succession of sequence elements they thought they had been pressing.

Upon entering the research lab, the participants were taken to a cubicle with a computer, on which the task was to be performed. There they signed the informed consent form and received written instructions for the experiment. The mobile phones of the participants were collected for the duration of the experiment. The participants were instructed to perform quickly but with as few errors as possible (preferably less than 8% per subblock). Error feedback at the end of each subblock consisted of the number of erroneous key presses divided by the total number of key presses in a subblock. These instructions were then presented also on the screen. Subsequently, the participants completed the 3 or 6 blocks of sequences. During the 4-minute breaks after each block, the participants were allowed to rest. Thereafter, they completed the awareness test on the computer in the presence of the experimenter during which the keyboard was covered to prevent participants from seeing the locations of the keys they had pressed.

The second part of the experiment took place on Day 2, which was always the day after Day 1. During this part, the participants carried out the test phase, and they then completed the same computerized awareness test as on Day 1. This always took place in the presence of the experimenter with the keyboard covered. On Day 1, Limited-Practice participants worked for 1 h, and Extended-Practice participants worked for 2 to 3 h. On Day 2, both groups worked for about 30 min.

#### Analyses

We generally reported only significant effects. Given that the practice blocks involved different numbers of trials in the Limited- and Extended-Practice groups, we analyzed as initial performance the first 12 trials (i.e., sequences) of Block 1 and as final performance during practice the last 12 trials in the last practice block (i.e., Block 3 or 6).^3^ This allowed a single ANOVA that allowed comparison of the means of the first and of the last 12 practice trials for each participant of the four (Limited/Extended-Practice × Low/High CI-Group) groups. The only difference between the two practice groups was the number of practice trials in between the first 12 and the last 12 practice trials. Error proportions consisted of the number of erroneous key presses divided by the actual number of key presses. These proportions were subjected to an arcsine transformation and then analyzed with the same ANOVA designs as response times (RTs) (Winer et al., 1991). The *p* values of the *F* tests in Experiments 1 and 2 were Greenhouse–Geisser corrected when Mauchly's test of sphericity was significant.

The analyses of the Awareness Tests were based on the numbers of correct responses of each reproduced sequence. As participants were free to decide which sequences to reproduce first, second and third, they could reproduce their 3 sequences in 6 different orders. Therefore, we first determined for each participant which of these 6 sequence orders yielded the highest number of correct responses. We then analyzed these results with nonparametric mixed ANOVAs using the nparLD package (Noguchi et al., 2012) in R Studio (R-Core_Team, 2020). As this software package does not allow more than either 2 within- or 2 between-subject variables (i.e., a F1 LD F2 or F2 LD F1 design), we used several simple ANOVA designs on subsets of the data and across data pooled over a few independent variables.

We used Pearson correlations between the numbers of correct responses in the Spatial and Verbal awareness tests and total execution time of the sequences in the four test conditions, indicated as ‘r’, and Spearman correlations between the numbers of correct responses in the Spatial and Verbal awareness tests and the proportions of errors in those conditions, indicated as ‘r_s_’.

### Results

#### Practice on day 1

The analyses of RTs and errors during practice involved 2 (CI-Group: BP vs. RP) × 2 (Practice-Group: Limited vs. Extended) × 2 (FirstLast12: first vs. last 12 trials during practice) × 4 (Sequence Position) mixed ANOVAs with CI-Group and Practice-Group as between-subject variables. RTs generally reduced with practice as evidenced by the FirstLast12 main effect (first 12 trials: 451 ms, last 12 trials: 272 ms), F(1,44) = 134.94, p < 0.001, η_p_^2^ = 0.75.

**Fig. 1:**
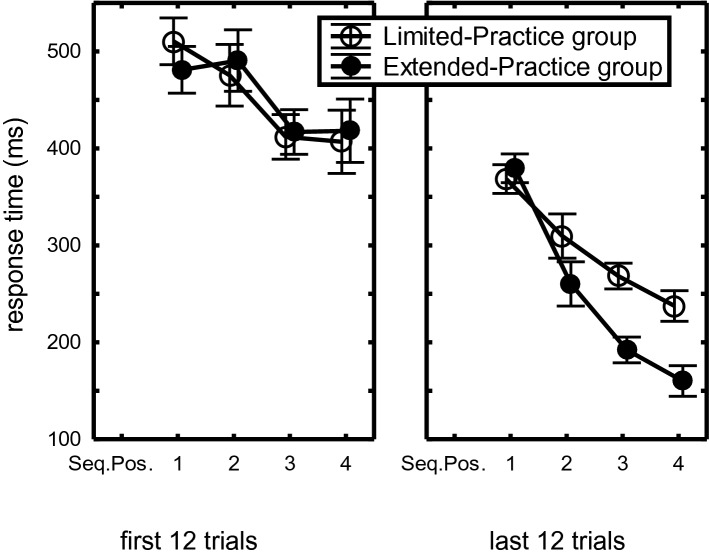
Mean response times in the first and last 12 trials of the Limited- and the Extended-Practice groups as a function of Sequence Position in the practice phase of Experiment 1. Here and in the figures below, the bars indicate the standard error of the means

The Sequence Position main effect showed an almost linear RT decrease with successive positions (435 ms, 384 ms, 322 ms, 306 ms, respectively), *F*(3,132) = 53.33, *p* < 0.001, η_p_^2^ = 0.55. The FirstLast12 × Sequence Position interaction indicated that the benefit of the last 12 over the first 12 practice trials increased with sequence position (122, 198, 184, 214 ms, respectively), *F*(3,132) = 8.54, *p* < 0.001, η_p_^2^ = 0.16. This effect was modulated by Practice-Group and CI. First, the FirstLast12 × Sequence Position × Practice-Group interaction, *F*(3,132) = 4.76, *p* = 0.003, η_p_^2^ = 0.10, indicated that later responses in the sequence benefited more from practice in the Extended- than in the Limited-Practice group (Fig. 1).

**Fig. 2:**
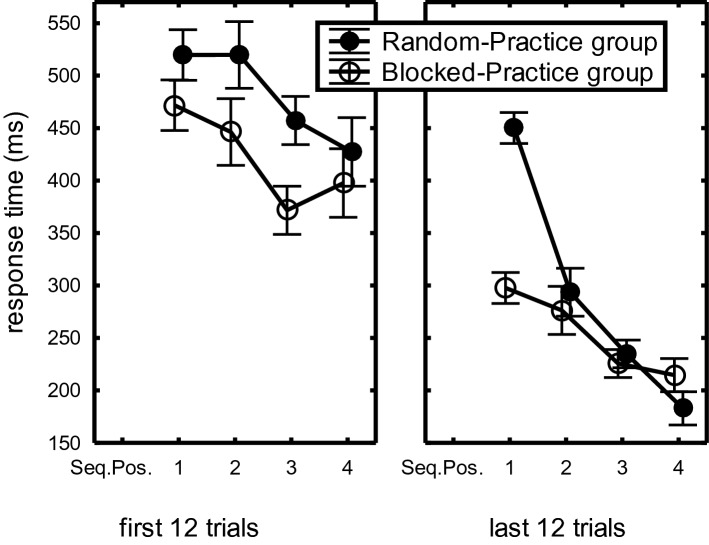
Response times in the first and last 12 trials of the Blocked- and Random-Practice groups across practice groups as a function of Sequence Position in the practice phase of Experiment 1

Second, a FirstLast12 × Sequence Position × CI-Group interaction, *F*(3,132) = 9.52, *p* < 0.001, η_p_^2^ = 0.18, together with a superseding Sequence Position × CI-Group interaction, *F*(3,132) = 6.48, *p* < 0.001, η_p_^2^ = 0.13, showed that in the first 12 practice trials there was a BP advantage at all four sequence positions (Fig. 2), but that in the last 12 practice trials this BP advantage increased at R_1_ and reduced at R_234_. This effect of practice was observed in the Limited- as well as the Extended-Practice groups.

Arcsine transformed error proportions in the first and last 12 practice trials were analyzed with the above ANOVA design and showed no significant effects. Error proportions amounted to 1.4% per sequence position on average.

### Test phase on day 2

#### Retention block

**Fig. 3:**
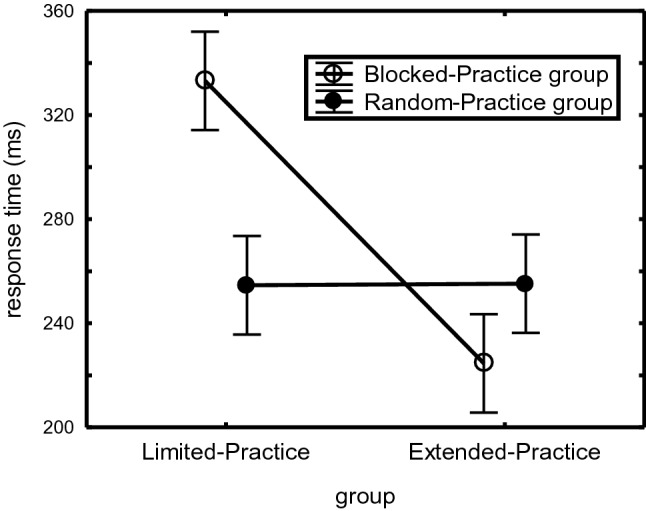
Mean RTs of the 4-key familiar sequences in the Retention condition of the test phase on Day 2 for the Limited- and Extended-Practice groups in “Experiment 1”, separately for the BP and RP groups. Standard error of the means are relatively large because they also include the Sequence position effects (cf. Figs. 1 and 2)

We used a 2 (Practice-Group) × 2 (CI-Group) × 3 (Sequence: 3 alternatives) × 4 (Sequence Position) ANOVA with Practice-Group and CI-Group as between-subjects variables to analyze the RTs in the Retention condition. It showed main effects of Practice-Group, *F*(1,44) = 8.14, *p* = 0.006, η_p_^2^ = 0.16, and Sequence Position, *F*(3,132) = 142.59, *p* < 0.001, η_p_^2^ = 0.76. More importantly, the Practice-Group × CI-Group interaction, *F*(1,44) = 8.31, *p* = 0.006, η_p_^2^ = 0.16, showed a significant CI-effect in the Limited-Practice group (79 ms), *F*(1,44) = 8.60, *p* = 0.005, η_p_^2^ = 0.16, while CI had no significant effect in the Extended-practice group (Fig. 3).

The CI-Group × Sequence Position interaction showed that the advantage of RP over BP in the retention test more or less increased with sequence position, *F*(3,132) = 4.13, *p* = 0.02, η_p_^2^ = 0.09, from − 11 ms at T_1_, 34 ms at T_2_ and 29 ms at T_3_, to 42 ms at T_4_. Planned comparisons showed that the CI-effect increased with sequence position only for the Limited-Practice group, *F*(3,132) = 2.87, *p* = 0.04, η_p_^2^ = 0.06 (CI-effect: 41, 104, 77, 92 ms, respectively).

The error analysis showed only a Sequence Position main effect, *F*(3,132) = 5.65, *p* = 0.001, η_p_^2^ = 0.11, indicating that error proportions increased from 0.4% at R_1_, to 2.0% for R_2_, and 1.6% for R_3_ and R_4_.

Hence, the Retention test showed that the CI-effect emerged after limited practice and not after extended practice, and that after limited practice the benefit of RP became stronger for later sequence positions.

### Overnight consolidation of familiar sequences

To assess whether performance changes differently after one night as a function of CI condition and amount of practice, we performed a 2 (Practice-Group) × 2 (CI-Group) × 2 (Day: Day 1-Last 12 trials vs. Day 2-Retention) × 3 (R_234_) mixed ANOVA with Practice-Group and CI-Group as between-subject variables (see earlier Analyses section for details). R_1_ was not included in this ANOVA because on Day 1 RP participants could not anticipate S_1_ while BP participants could.

**Fig. 4:**
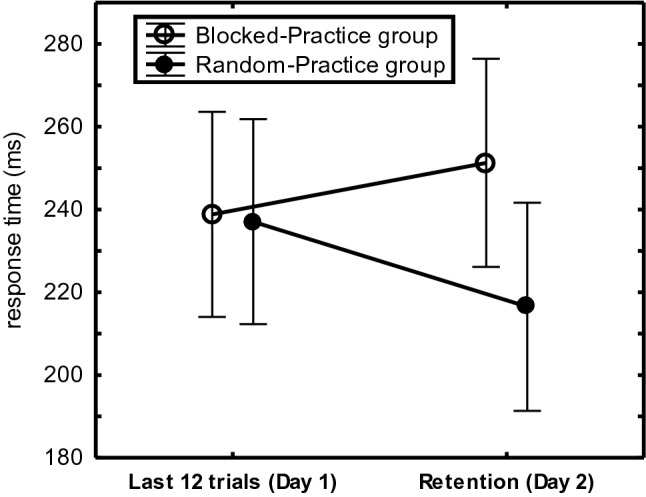
Overnight consolidation of sequence execution rate (R_234_) in “Experiment 1” across practice groups in the last 12 practice trials on Day 1 versus the Retention phase on Day 2 in the RP- and BP participants

Overnight consolidation was indicated for RP participants and not for BP participants by the CI-Group x Day interaction, *F*(1,44) = 5.37, *p* = 0.03, η_p_^2^ = 0.11. As depicted in Fig. 4, RP participants executed R_234_ faster on Day 2 than on Day 1 (217 ms vs. 237 ms: 20 ms) whereas BP participants executed R_234_ slower on Day 2 than on Day 1 (239 ms vs. 251 ms: -12 ms consolidation; total consolidation effect: 32 ms). This improvement due to overnight consolidation occurred in the Limited-Practice and the Extended-Practice participants (RP overnight benefit minus BP overnight benefit: 39 ms vs. 27 ms, respectively).

### Novel and unstructured conditions

A 2 (Practice-Group) × 2 (CI-Group) × 2 (Sequence: Novel vs. Unstructured) × 4 (Sequence Position) mixed ANOVA showed by way of a Practice-Group main effect that the Extended-Practice group was 43 ms faster than the Limited-Practice group on these novel and unstructured sequences, *F*(1,44) = 4.67, *p* = 0.04, η_p_^2^ = 0.10. A Practice-Group × CI-Group crossover interaction across the unstructured and novel sequences, *F*(1,44) = 16.79, *p* < 0.001, η_p_^2^ = 0.28, indicated that in the Limited-Practice group RTs were shorter for RP than for BP participants while in the Extended-Practice group RTs were significantly longer in RP participants than in BP participants. The same crossover interaction was significant for just the Unstructured sequences, *F*(1,44) = 6.49, *p* = 0.01, η_p_^2^ = 0.13, and for just the novel sequences, *F*(1,44) = 21.89, *p* < 0.001, η_p_^2^ = 0.33. For the Limited-Practice group, the RP advantage over BP was significant in the Unstructured condition (59 ms), *F*(1,44) = 5.23, *p* = 0.03, η_p_^2^ = 0.11, and the Novel condition (91 ms), *F*(1,44) = 14.83, p < 0.001, η_p_^2^ = 0.25. For the Extended practice group, the BP advantage was significant in the Novel condition (65 ms), *F*(1,44) = 7.65, *p* = 0.008, but not in the Unstructured condition (34 ms), *F*(1,44) = 1.73, *p* = 0.20.

**Fig. 5:**
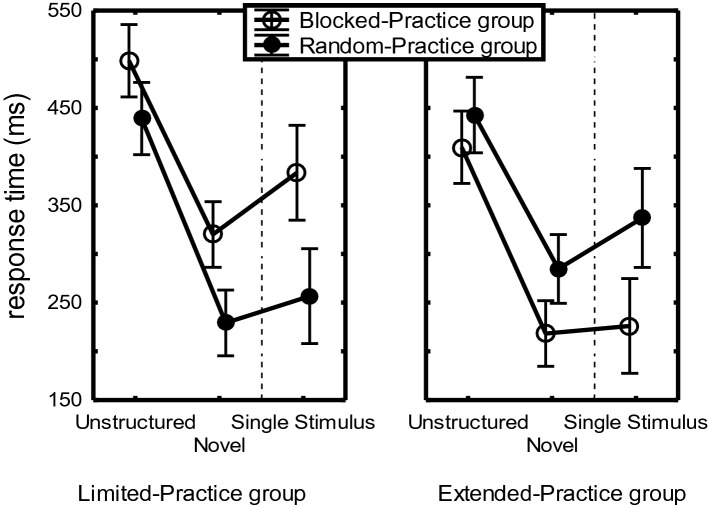
Mean RTs for the 4-key sequences in the Unstructured and the Novel conditions, as well as the subsequently performed Single-stimulus condition of the test phase of Experiment 1 (indicated by the dashed line), for each of the four participants groups. The CI-effect consists of the RT difference between RP and BP participants, and is fact negative in the Extended-Practice group. The dashed line indicates that the Single-stimulus analysis is reported separately

In the Limited-Practice group (Fig. 5), the RP benefit appeared significant when tested separately for both the Unstructured and the Novel sequences (benefit: 59 vs. 91 ms, resp.), *F*s(1,44) > 5.23, *p*s < 0.03, η_p_^2^s > 0.11. In the Extended-Practice group, the advantage of BP over RP was significant only with the Novel Sequence, *F*(1,44) = 7.65, *p* = 0.008, η_p_^2^ = 0.15 (Fig. 5).

The Sequence Position main effect showed that RTs reduced with sequence position (404, 347, 345, and 326 ms, resp.), *F*(3,132) = 38.77, *p* < 0.001, η_p_^2^ = 0.47, and according to a Sequence × Sequence Position interaction, *F*(3,132) = 28.83, *p* < 0.001, η_p_^2^ = 0.40, this reduction with sequence position was more pronounced for Novel than for Unstructured sequences. A CI-Group × Sequence Position interaction, *F*(3,132) = 2.66, *p* = 0.05, η_p_^2^ = 0.06, showed that across Novel and Unstructured sequences the benefit of RP over BP increased with sequence position.

Planned comparisons to test whether the RP advantage over BP would hold especially for sequence execution (i.e., R_234_) in the Limited-Practice group confirmed this for the Novel condition (29 ms, 137 ms, 99 ms, 99 ms, resp.), *F*(3,132) = 3.71, *p* = 0.01, η_p_^2^ = 0.08, but not for the Unstructured condition (35, 55, 69, 78 ms, respectively), *F*(3,132) = 2.45, *p* = 0.07, η_p_^2^ = 0.05. The BP advantage did not change with sequence position in the Unstructured and Novel conditions in the Extended-Practice group.

The error analysis showed only a Sequence Position main effect, *F*(3,132) = 14.62, *p* < 0.001, η_p_^2^ = 0.25, this time indicating an error proportion increase from 0.6% at R_1_, to 1.5% for R_2_, 1.8% for R_3_, and 2.7% at R_4_.

So, a CI-like effect was found after limited practice in the Novel and the Unstructured conditions. In the Novel condition, this effect concerned primarily R_234_ while it was not different for the 4 sequence positions in the Unstructured condition. Instead, after extended practice BP participants were faster than RP participants in the Novel condition (but not in the Unstructured condition).

### Single-stimulus condition

One participant in the Extended-Practice group did not reproduce any of the three sequences correctly in response to just S_1_. The RTs obtained with correct sequences in the Single-stimulus condition for the remaining participants were analyzed with a 2 (Practice-Group) × 2 (CI-Group) × 3 (Sequence) × 4 (Sequence Position) ANOVA. It showed a Sequence Position main effect indicating that RTs reduced with sequence position (386, 342, 245, 229 ms, respectively), *F*(3,129) = 60.97, *p* < 0.001, η_p_^2^ = 0.59.

As depicted in Fig. 5, the Single-stimulus condition also showed the interaction between Practice-Group and CI-Group that was observed in the other test conditions, *F*(1,43) = 23.34, *p* < 0.001, η_p_^2^ = 0.35. It reflected for the Limited-Practice group that RP participants were again significantly faster than BP participants (RP: 257 ms vs. BP: 383 ms), *F*(1,43) = 13.57, *p* < 0.001, η_p_^2^ = 0.24. Conversely, in the Extended-Practice group BP participants again executed the sequences in the Single-stimulus condition faster than RP participants (BP: 226 ms vs. RP: 337 ms), *F*(1,43) = 9.95, *p* = 0.003, η_p_^2^ = 0.19.

The ANOVA on error proportions showed a main Sequence Position effect indicating that error proportion varied across the 4 key presses, R_1_: 1.0%, R_2_: 8.9%. R_3_: 6.9%, and R_4_: 3.6%, *F*(3,132) = 46.58, *p* < 0.001, η_p_^2^ = 0.51.

In short, the Limited-Practice group again showed at all sequence positions the CI-effect in the Single-stimulus condition while the Extended-Practice group showed the reverse, namely an advantage of BP over RP participants.

### The CI-effect across the test conditions

To compare the size of the CI-effect amongst the four test conditions, we used a 2 (Practice-Group) × 2 (CI-Group) × 4 (Sequence: Retention, Novel, Unstructured, Single-stimulus) × 4 (Sequence Position) mixed ANOVA. Planned comparisons involving just the Limited-Practice group showed that the RP benefit was 127 ms larger in the Single-Stimulus condition than in the Unstructured condition (59 ms), *F*(1,43) = 6.03, *p* = 0.02, η_p_^2^ = 0.12, and that tended to be larger also than in the Retention (79 ms) and the Novel conditions (91 ms), *F*(1,43) = 3.67, *p* = 0.06, η_p_^2^ = 0.08. The RP benefit in the Limited-Practice group was not different across the Novel and Unstructured conditions.

Evaluating the effect of amount of practice on the test conditions showed that RTs were shorter for the Extended- than for the Limited-Practice group in the Retention condition (241 ms vs. 294 ms), *F*(1,43) = 7.44, *p* = 0.009, η_p_^2^ = 0.15, and the Unstructured condition (426 ms vs. 469 ms), *F*s(1,43) = 5.16, *p*s = 0.03, η_p_^2^ = 0.11, but the effect of extended practice did not reach statistical significance for the Novel condition (251 ms vs. 275 ms), *F*(1,43) = 1.85, *p* = 0.18, and the Single-stimulus conditions (282 ms vs. 320 ms), *F*s(1,43) = 2.44, *p* = 0.13. This larger benefit of extended over limited practice in the Retention than in the Novel condition was itself significant, *F*(1,43) = 4.40, *p* = 0.04, η_p_^2^ = 0.09.

### Awareness task

The sequences were reproduced more often entirely correct in the Spatial than in the Verbal awareness test (49% vs. 23% of the total of 288 sequences in each task), and this was not very different for the Limited and the Extended-Practice groups (104 and 103 correct of 288 sequences, yielding 36% in both groups), and for the BP and RP participants (both 36% too).

The total numbers of correct responses were analyzed across the 2 practice groups, the 4 sequence positions and the 3 sequences with non-parametric 2 (Day) × 2 (CI-Group) × 2 (Test: Verbal vs. Spatial) mixed ANOVAs with CI-Group as between-subject variable (this type of ANOVA does not allow a full design). This analysis confirmed that the Spatial awareness test was performed better than the Verbal awareness test (64% vs. 46% correct responses), ATS(1) = 62.60, *p* < 0.001.

Performance had improved on Day 2, which may be attributed to the participants’ experience with the awareness test on Day 1, ATS(1) = 14.76, *p* < 0.001. Still, a CI-Group x Day interaction, ATS(1) = 6.48, *p* = 0.01, indicated greater improvement of explicit sequence knowledge on Day 2 for RP (Day 1: 47%, Day 2: 61% correct) than for BP participants (Day 1: 55%, Day 2: 58% correct), yielding similar performance on Day 2. This greater improvement from Day 1 to Day 2 for RP than BP participants across the Spatial and Verbal task was significant only for the Limited-Practice group (BP: from 50 to 51% correct; RP: from 49 to 67%), ATS(1) = 7.81, *p* = 0.005, but not for the Extended-Practice group (BP: from 60 to 65% correct; RP: from 45 to 55%), ATS(1) = 0.54, *p* = 0.70.

Correlations between the awareness tests and sequence execution times showed that participants with more spatial awareness executed sequences faster in the Novel, *r*(48) = − 37, *p* = 0.01, Unstructured *r*(48) = − 0.30, *p* = 0.04), and Single-stimulus *r*(47) = − 0.39, *p* = 0.01. BP participants showing more spatial awareness executed sequences faster in the Retention *r*(24) = − 0.48, *p* = 0.02, Novel *r*(24) = − 0.45, *p* = 0.02, and Unstructured sequence conditions *r*(24) = − 0.45, *p* = 0.03, while these correlations were not significant for RP participants. Participants in the Extended-Practice group showing more verbal awareness executed sequences faster in the Retention condition, *r*(*N* = 24) = − 0.44, *p* = 0.005. Error rate in the Single-stimulus condition was lower for BP participants with a higher spatial and verbal awareness, *r*_s_*s*(*N* = 24) < − 0.41, *p*s < 0.05, and for participants with more verbal awareness of the Limited-Practice group, *r*_s_*s*(*N* = 24) = − 0.46, *p*s < 0.03.

In short, all 4 (Limited/Extended-Practice × Low/High CI-Group) groups showed the same proportions of participants with full sequence knowledge and this involved mostly spatial sequence knowledge. In the Limited-Practice group, spatial and verbal awareness showed more overnight improvement for RP than BP participants. Spatially more aware participants, especially BP participants, executed not only familiar (Retention, Single-stimulus), but also unfamiliar sequences (Unstructured, Novel) faster. Verbal awareness was associated in Extended-Practice participants with faster sequence execution in the Retention condition, and with less errors in the Single-stimulus condition for the BP and the Limited-Practice participants.

### Discussion

Experiment 1 confirmed the hypothesis that for the 4-key sequences that can be prepared in short-term memory as a whole, the CI-effect occurs with limited and not with extended practice (Fig. 3). It also confirmed that this benefit of RP over BP can be attributed to overnight consolidation (Fig. 4) (Kim & Wright, 2020; Lin et al., 2011). This retention advantage of RP after limited practice of 4-key sequences was found despite the use of a blocked protocol in the Retention condition on Day 2 that could have benefited BP participants. The test phase indicated that this retention benefit of limited practice RP is most likely based on a combination of enhanced sequence representations (improving performance in the Retention and Single-stimulus conditions), general sequencing skill (improving the Novel sequence), and reaction skill (improving the Unstructured sequence). Correlations between awareness and execution errors suggest that performance in the Single-stimulus condition involved explicit sequence representations. The limited awareness of Extended-Practice RP participants (55% correct in the awareness test) may then explain the low execution rate of these participants in the Single-stimulus condition (Fig. 5). The finding that more aware Limited-Practice RP participants were faster on the Novel and Unstructured sequences suggests that these participants were not faster on the practiced sequences because they applied their explicit sequence knowledge, but because they possessed superior processing skills. Finally, extended practice seems to have induced a general preparation skill that allowed BP participants of the Extended-Practice group to execute sequences in the blocked Single-stimulus and Novel conditions faster than RP participants.

## Experiment 2

Experiment 2 was identical to Experiment 1 except that it involved 7-key sequences. As explained in the Introduction we expected that learning 7-key sequences would improve during BP, perhaps to the level of RP, because these sequences cannot be entirely prepared in short-term memory. This would require preparation of successive segments in a single sequence and increase CI, even in BP (Abrahamse et al., 2013; Verwey, 2001). We used a version of the 7-key sequence that was recently found to show a spontaneous concatenation point at the fifth response across all participants (Verwey & Dronkers, 2019). We further explored whether overnight consolidation can be observed also in the various practice groups practicing these 7-key sequences, and whether the development of the skills and representations in Table 1 benefits from extended practice.

### Method

The main difference with Experiment 1 involved adjusting the software of Experiment 1 to use 7-key sequences (i.e., VCBNCVN, NVCBVNB, BNVCNBC, and CBNVBCV, used also in Verwey & Dronkers, 2019). These sequences were balanced across the practice and test phases so that across participants each sequence occurred equally often as the novel sequence in the test phase and as one of the three familiar sequences in the practice phase. The Day 1 session lasted about 2.5 to 3.5 h for the Extended-Practice group, and about 40 min for the Limited-Practice group. The Day 2 test session lasted approximately 30 min for both groups. In addition, we used newer computers (Dell Optiplex 7050) running Windows 10 Enterprise. These computers were equipped with a 144 Hz AOC Freesync monitor and a PS/2 keyboard.

The 48 right-handed participants were between 18 and 30 years old (mean 22, Extended-Practice group: 7 males; Limited-Practice group: 11 males). All were students of the University of Twente who had no previous experience with the DSP task. They participated voluntarily or in exchange for course credits and indicated no to smoke and that they had not consumed alcohol in the preceding 24 h. They were randomly assigned to the four groups. All participants filled out an informed consent.

### Results

#### Practice on Day 1

The analyses of RTs and errors during practice involved 2 (CI-Group: BP vs. RP) × 2 (Practice-Group: Limited vs. Extended) × 2 (FirstLast12: first vs. last 12 trials during practice) × 7 (Sequence Position) mixed ANOVAs with CI-Group and Practice-Group as between-subjects variables. RTs reduced with practice as evidenced by the FirstLast12 main effect (from 459 to 311 ms), *F*(1,44) = 247.27, *p* < 0.001, η_p_^2^ = 0.85, and more so for the Extended than Limited-Practice group, *F*(1,44) = 18.61, *p* < 0.001, η_p_^2^ = 0.30. The Practice-Group x FirstLast12 interaction showed that across all sequence positions, RTs reduced more during practice for the Extended than the Limited-Practice group, *F*(1,44) = 9.70, *p* = 0.003, η_p_^2^ = 0.18.

**Fig. 6:**
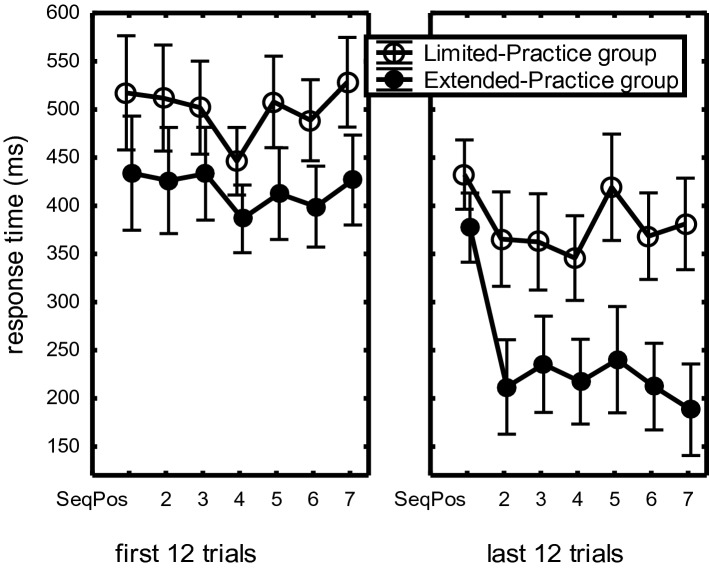
Response times in the first and last 12 trials of the Limited- and the Extended-Practice groups as a function of Sequence Position in the practice phase of Experiment 2

The larger RT reduction in the Extended than in the Limited-Practice group was modulated by Sequence Position as indicated by a Practice-Group x FirstLast12 x Sequence Position interaction, *F*(6,264) = 3.00, *p* = 0.02, η_p_^2^ = 0.06 (Fig. 6), and the superseding Sequence Position main effect, *F*(6,264) = 11.70, η_p_^2^ = 0.21, the Sequence Position x Practice-Group interaction, *F*(6,264) = 2.67, *p* = 0.04, η_p_^2^ = 0.06, and the Sequence Position x FirstLast12 interaction, *F*(6,264) = 12.54, *p* < 0.001, η_p_^2^ = 0.22. The notion that these interactions with sequence position were caused especially by the deviating effect of practice on R_1_ in the Extended-Practice group (see right frame of Fig. 6) was confirmed by a contrast analysis consisting of a Practice-Group × FirstLast12 × T_1_ vs T_234567_ interaction, *F*(1,44) = 11.90, *p* = 0.001, η_p_^2^ = 0.21, indicating that these interactions with Sequence Position can indeed be attributed to the small RT difference at R_1_ and the large difference at R_234567_ in the last 12 trials. Planned comparison confirmed that execution rate (R_234567_) was faster in the last 12 practice trials for the Extended- than the Limited-Practice group, *F*(1,44) = 29.2, *p* < 0.001, η_p_^2^ = 0.40.

Planned comparisons showed that in the last 12 practice trials of the Limited-Practice group R_5_ was 55 ms slower than R_23467_, *F*(1,44) = 8.95, *p* = 0.005, η_p_^2^ = 0.05. This R_5_ slowing was significant for just BP-participants (67 ms slower), *F*(1,44) = 6.67, *p* = 0.01, η_p_^2^ = 0.13, and was not significant for the group of RP participants (43 ms slower), *F*(1,44) = 2.72, *p* = 0.11. So, during the first 24 practice trials per sequence R_5_ had become relatively slow, but this R_5_ slowing had reduced to about half that size after 504 practice trials.

The error ANOVA showed two significant interactions, namely the FirstLast12 × Sequence Position interaction, *F*(6,264) = 3.56, *p* = 0.003, η_p_^2^ = 0.07, and the FirstLast12 × Sequence Position × CI-Group interaction, *F*(6,264) = 2.57, *p* = 0.02, η_p_^2^ = 0.06. These interactions were caused by an especially high error rate for R_123_ in the first 12 trials of the RP participants (2.8%, 3.3%, 3.0%, respectively; R_4567_ in that condition < 1.1%), and for R_6_ in the last 12 trials (in BP as well as RP: 2.9%, 3.0%, respectively; others < 1.6%).

In short, sequence execution rate reduced more with practice in the Extended- than in the Limited-Practice group. R_5_ was relatively slow at the end of practice, and this reached statistical significance for the Limited- but not for the Extended-Practice group. Comparing BP and RP participants showed no significant RT differences, but RP showed more errors in R_123_ in the first 12 practice trials.

### Test phase on Day 2

#### Retention

**Fig. 7:**
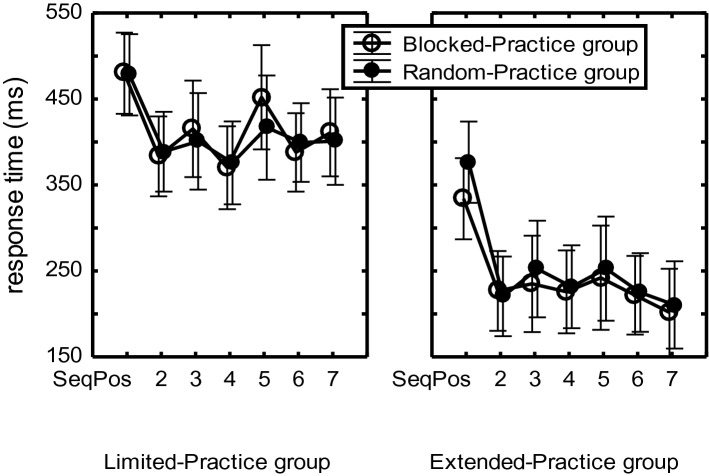
Response times in the Retention condition of the test phase of Experiment 2 as a function of Practice-Group, Contextual Interference and sequence position

We used a 2 (Practice-Group) × 2 (CI-Group) × 3 (Sequence) × 7 (Sequence Position) mixed ANOVA to analyze RTs and errors in the Retention condition on Day 2 with Practice-Group and CI-Group as between-subjects variables. It showed by way of a Practice-Group main effect, *F*(1,44) = 38.37, *p* < 0.001, η_p_^2^ = 0.47, that mean RT was shorter for the Extended- than for Limited-Practice group. All effects associated with CI-Group including its main effect were far from significant (*p*s > 0.39), even in just the Limited-Practice group (414 ms vs. 409 ms), *F*(1,22) = 0.15, *p* = 0.71 (Fig. 7).

A Sequence Position main effect showed the typical difference between RTs at the various sequence positions, and especially that R_1_ was much slower (417 ms for R_1_, vs. < 324 ms for R_234567_), *F*(6,264) = 36.39, *p* < 0.001, η_p_^2^ = 0.45. A Sequence Position x Practice-Group interaction indicated that this sequence position effect was different for the two Practice-Groups, *F*(6,264) = 3.42, *p* = 0.01, η_p_^2^ = 0.07 (Fig. 7). A planned comparison of T_5_ and T_23467_ was significant across practice and CI-Groups, *F*(1,44) = 11.47* p* = 0.001, η_p_^2^ = 0.21. It was significant also for the Limited-Practice group (T_5_–T_23467_ = 41 ms), *F*(1,44) = 9.63, *p* = 0.003, η_p_^2^ = 0.18, but not for the Extended-Practice group (T_5_–T_23467_ = 22 ms), *F*(1,44) = 2.85, *p* = 0.10.

The aforementioned design was used also for an ANOVA on arcsine transformed error proportions per response. It showed by way of a CI-Group × Practice-Group interaction, *F*(1,44) = 4.19, *p* = 0.05, η_p_^2^ = 0.09, that error frequency was similar in the Limited-Practice group for BP participants and RP participants (1.6% vs. 1.3%, resp.), while in the Extended-Practice group it was smaller for BP participants than for RP participants (0.7% vs. 1.6%, resp.). Furthermore, a Sequence Position main effect showed that error rate was relatively low for R_1_, R_4_ and R_7_ compared with the other responses (0.4%, 1.5%, 2.0%, 0.5%, 2.0%, 1.9%, and 1.2%, resp), *F*(6,264) = 5.29, *p* < 0.001, η_p_^2^ = 0.09.

Hence, during retention sequence execution was clearly faster after extended than after limited practice but no difference was found between participants who had been practicing in BP and RP. Concatenation was again observed in that R_5_ was relatively slow for the Limited-Practice group, and this R_5_ slowing was smaller and not significant for the Extended-Practice group.

#### Overnight consolidation of familiar sequences

**Fig. 8:**
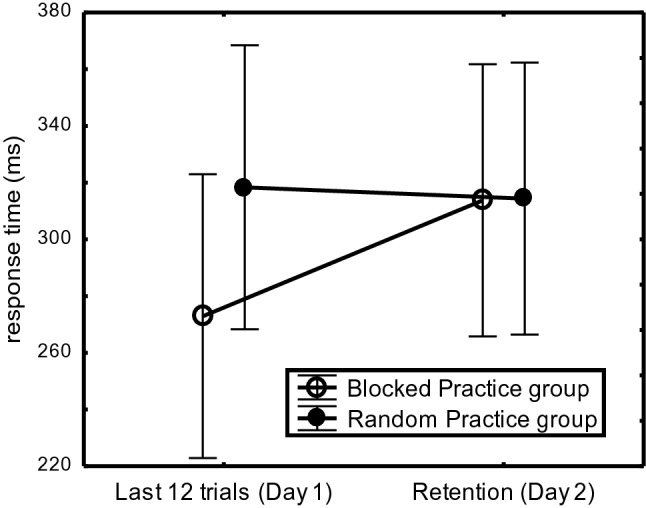
Execution rate (R_234567_) in the final 12 practice trials of Day 1 and in the Retention phase on Day 2. Bars indicate standard errors of the mean

Overnight consolidation was assessed as a function of CI condition and amount of practice with a 2 (Practice-Group) × 2 (CI-Group: BP vs. RP) × 2 (Day: Day 1-Last 12 trials vs. Day 2-Retention) × 6 (R_2-7_) mixed ANOVA with Practice-Group and CI-Group as between-subject variables (R_1_ was again excluded). This ANOVA showed a Day × CI interaction, *F*(1,44) = 8.86, *p* = 0.004, η_p_^2^ = 0.17, indicating that the advantage of BP over RP participants observed for R_234567_ at the end of Day 1 was eliminated during Retention on Day 2 (Fig. 8). This effect indicated that BP participants executed the sequences slower on Day 2 than at the end of Day 1 (273 ms vs. 314 ms: 41 ms) whereas RP participants executed R_2-7_ as fast on Day 2 as on Day 1 (318 ms vs. 314 ms: − 4 ms consolidation; total consolidation effect: 45 ms). This effect was equally large in the Limited- and the Extended-Practice participants (total consolidation: 62 ms vs. 27 ms, respectively),* F*(1,44) = 1.38, p = 0.25. Hence, in both practice groups the execution rate slowing after a night’s rest was observed with the BP participants and not with RP participants.

#### Novel and unstructured sequences

RTs in the unstructured and novel sequences were analyzed using a 2 (Practice-Group) × 2 (CI-Group) × 2 (Sequence: Unstructured vs. Novel) × 7 (Sequence Position) mixed ANOVA with Practice-Group and CI-Group as between-subject variables. CI-Group was not significant as a main effect, *F*(1,44) = 0.89, p = 0.35, and neither did any of the interactions with CI-Group reach significance, *p*s > 0.14.

**Fig. 9:**
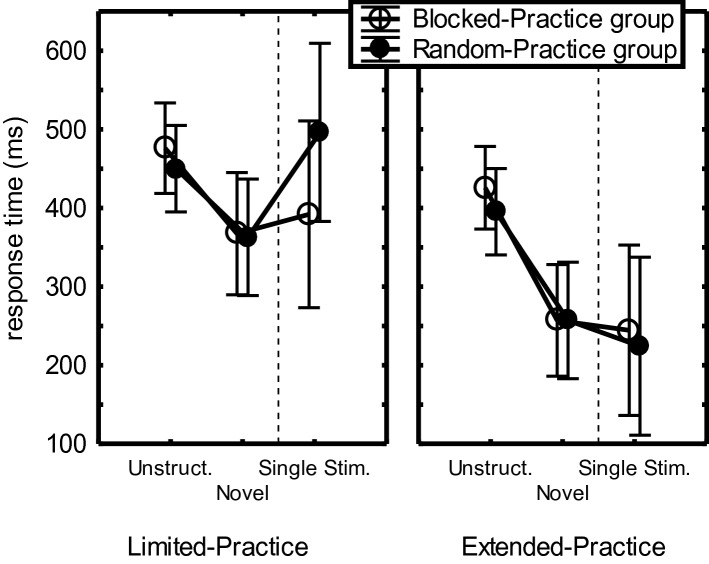
Mean RTs for the 7-key sequences in the Unstructured and the Novel conditions, as well as in the subsequently performed Single-stimulus condition of the test phase of Experiment 2. The dashed line indicates that the Single-stimulus condition followed the block containing the Unstructured and Novel conditions and was analyzed separately

The main Practice-Group effect showed that RTs were shorter in the Extended-Practice group than in the Limited-Practice group, *F*(1,44) = 13.02, *p* < 0.001, η_p_^2^ = 0.23 (cf. Figure 9). The Sequence main effect indicated that sequences were executed faster in the Novel than in the Unstructured condition (320 ms vs. 441 ms), *F*(1,44) = 229.83, *p* < 0.001, η_p_^2^ = 0.84. The advantage of the novel over the unstructured condition was larger for the Extended-Practice group than for the Limited-Practice group, *F*(1,44) = 12.68, *p* < 0.001, η_p_^2^ = 0.22. Still, the RT advantage of the Extended over the Limited-Practice group was significant for both the Unstructured and the Novel conditions, *F*s(1,44) > 7.36, *p*s < 0.009, η_p_^2^s > 14.

The error analysis involving the same ANOVA showed a Sequence main effect indicating that error rate was higher for Unstructured than for Novel sequences (1.8% vs. 1.6% per sequence position), *F*(1,44) = 6.12, *p* = 0.02, η_p_^2^ = 0.12. Error rate generally increased with sequence position, from 0.6% at R_1_ to 2.1% per sequence position at R_7_, with a 2.4% peak at R_3_, *F*(6,264) = 4.71, *p* < 0.001, η_p_^2^ = 0.10.

In short, unstructured sequences and, even more, novel sequences were executed faster after extended practice of the familiar sequences than after limited practice, but this was not different for BP and RP participants.

#### Single-stimulus condition

In the Limited-Practice group, 6 of the 12 BP participants and 8 of the 12 RP participants were not able to execute any of their 3 sequences in the Single-stimulus condition without external guidance. In the Extended-Practice group, this was the case with 1 BP and 3 RP participants. The arcsine transformed proportions of accurately reproduced sequences (out of a maximum of 12 for each of the 3 sequences) were analyzed with a 2 (Practice-Group) × 2 (CI-Group) between-subjects analysis. It showed that the Extended-Practice group executed more sequences accurately than the Limited-Practice group (66.7% vs. 26.8% of the 3 × 12 = total 36 sequences per participant in the Single-stimulus block), *F*(1,44) = 29.1, *p* < 0.001, η_p_^2^ = 0.40.

Of the participants who had at least once executed correctly each of their 3 sequences in the Single-stimulus condition, mean RTs were analyzed using a 2 (Practice-Group) × 2 (CI-Group) × 3 (Sequence) × 7 (Sequence Position) mixed ANOVA. This ANOVA showed that participants in the Extended-Practice group had shorter RTs than in the Limited-Practice group (277 ms vs. 600 ms), *F*(1,26) = 25.58, *p* < 0.001, η_p_^2^ = 0.50. Sequence Position had its usual main effect, *F*(6,156) = 7.54, *p* < 0.001, η_p_^2^ = 0.20.

In contrast to the suggestion in Fig. 9, RP participants were not slower than BP participants in the Limited- and neither in the Extended-Practice group, *F*(1,26) = 2.43, *p* = 0.13. However, in the Limited-Practice group this slowing was significant for R_567_, and more for RP than BP participants. This was indicated by a Practice-Group × CI-Group × Sequence Position interaction, *F*(6,156) = 2.49, *p* = 0.05, η_p_^2^ = 0.09, together with the superseding Practice-Group × Sequence Position, *F*(6,156) = 6.54, *p* < 0.001, η_p_^2^ = 0.22, and CI-Group × Sequence Position interactions, *F*(6,156) = 2.36, *p* = 0.03, η_p_^2^ = 0.08. Planned comparisons showed that R_567_ were significantly slower than R_234_ only for the Limited-Practice group (672 vs. 531 ms), *F*(1,26) = 25.94, *p* < 0.001, η_p_^2^ = 0.50, thus yielding a significant Practice-Group × Sequence segment (R_234_ vs. R_567_) interaction, *F*(1,26) = 22.11, *p* < 0.001, η_p_^2^ = 0.46. In the Limited-Practice group, this slowing at the end of the sequences was significant for the RP participants (T_234_: 605 ms, T_567_: 780 ms), *F*(1,26) = 24.65, *p* < 0.001, η_p_^2^ = 0.49, but did not reach significance for the BP participants (T_234_: 496 ms, T_567_: 548 ms), *F*(1,26) = 2.94, *p* < 0.10. This slowing of R_567_ relative to R_234_ was significantly larger in RP than in BP participants, *F*(1,26) = 10.34, *p* = 0.003, η_p_^2^ = 0.28. Finally, in the Extended-Practice group, R_5_ was slow relative to R_23467_, (290 vs 243 ms, resp.) *F*(1,26) = 4.54, *p* = 0.04, η_p_^2^ = 0.15.

The ANOVA with the above design on the arcsine transformed proportions correct key presses involved only participants who had been able to execute at least one of each of the three sequences accurately. It showed that less errors were made in the Extended- than in the Limited-Practice group (6.8% vs. 14.5%), *F*(1,34) = 11.85, *p* = 0.001, η_p_^2^ = 0.26, and that error rate monotonically increased between Sequence Positions 1 to 6, with a final reduction at Position 7 (from 5.5 to 17.6%, and then 15.3%), *F*(6,204) = 11.06, *p* < 0.001, η_p_^2^ = 0.26. According to a Practice-Group *, p* Sequence Position interaction, *F*(6,204) = 2.60, *p* = 0.05, η_p_^2^ = 0.06, this error rate increase until R_6_ was more pronounced for the Limited-Practice (from 6.0 to 24.3%) than for the Extended-Practice group (from 5.0 to 9.6%).

Taken together, Limited-Practice participants were able to produce only 27% of the sequences correctly in the Single-stimulus condition, and even Extended-Practice participants successfully produced only 67%. In the Limited-Practice group, R_567_ were slower than R_234_ and more so for RP than BP participants. After extended practice the familiar sequences were executed much faster. They showed no slowing of the last few responses but did show a relatively slow R_5_.

#### The CI-effect across the test conditions

A 2 (Practice-Group) × 2 (CI-Group) × 2 (Sequence: Retention, Novel, Unstructured, Single-stimulus) × 7 (Sequence Position) mixed ANOVA was performed to assess whether execution rates differed amongst the four test conditions. A planned Practice-Group × Retention vs. Unstructured interaction, *F*(1,40) = 58.75, *p* < 0.001, η_p_^2^ = 0.59, and a planned Practice-Group × Novel vs. Unstructured interaction, *F*(1,40) = 10.86, *p* = 0.002, η_p_^2^ = 0.21, showed that the effect of extended practice on Day 1 was substantially smaller on Day 2 in the Unstructured (410 ms vs. 463 ms) than in the Retention (240 vs. 402 ms) and Novel sequence conditions (257 ms vs. 365 ms). Likewise, a Practice-Group × Retention vs. Novel interaction, *F*(1,40) = 17.72, *p* < 0.001, η_p_^2^ = 0.31, showed that extended practice reduced RTs more for Retention than for Novel sequences. Also, extended practice had a greater effect on the Novel than on the Unstructured condition, *F*(1,40) = 10.86, *p* = 0.002, η_p_^2^ = 0.21. So, extended practice fastened execution in all test conditions, but the effect was largest for the Retention and the Single-stimulus conditions, smaller for the Novel, and smallest for the Unstructured condition.

#### Awareness task

Across the awareness tests on Days 1 and 2, the Extended-Practice group correctly reproduced 31% of the 7-key sequences (90 of the 288 sequences) while this amounted to only 6% (18 of 288) for the Limited-Practice group. BP participants had 26% correct sequences and RP participants 12%. There were 29% correct sequences for the Spatial awareness test and 12% for the Verbal awareness test, and 14% correct sequences on Day 1 and 23% on Day 2.

The non-parametric 2 (Day) × 2 (CI) × 2 (Test: Verbal vs. Spatial) mixed ANOVA with CI as between-subject variable on numbers of correct responses per sequence position showed more correct responses in the Spatial than in the Verbal awareness test (58% vs. 47%), ATS(1) = 9.49, p = 0.002, in BP than in RP participants (also 58% vs. 47%, resp.), ATS(1) = 7.29, *p* = 0.007, and on Day 2 than on Day 1 (56% vs. 49%), ATS(1) = 13.59, *p* < 0.001.

Across all participants, more Spatial awareness was associated with faster sequence execution in the Retention, *r*(*N* = 48) = − 0.56, *p* < 0.001, the Novel conditions, *r*(*N* = 48) = − 0.46, *p*s < 0.001, and the Single-stimulus condition, *r*(*N* = 44) = − 0.64, *p* < 0.001, but not in the Unstructured condition. This higher execution rate of more aware participants was observed also for each CI-Group in the Retention condition, *r*s(*N* = 24) > − 0.48, *p*s > 0.02, and the Single-stimulus condition, *r*s(*N* = 22) > 0.64, *p*s = 0.001. Separate tests for the Limited- and Extended-Practice groups showed significant correlations only for the Extended-Practice group, which showed after extended practice participants with more spatial awareness had with shorter execution times in the Retention and the Novel conditions, *r*s(*N* = 24) > − 0.43, *p*s < 0.04, and this was the case for all participants in the Single-stimulus condition, *r*(*N* = 44) = − 0.53, *p* = 0.01.

Less errors were made in the Single-stimulus condition by participants with more spatially awareness, *r*_s_(*N* = 38) = − 0.69, *p* < 0.001, and more verbal awareness, *r*_s_(*N* = 38) = − 0.37, *p* = 0.02. This correlation between execution errors and awareness was significant also for BP participants (spatial test: *r*(*N* = 21) = − 0.70, *p* < 0.001; verbal test: *r*(*N* = 21) = − 0.50, *p* = 0.02), and for RP participants for spatial awareness, *r*_s_(*N* = 17) = − 0.60, *p* = 0.01. These correlations were significant also when tested for just the Extended-Practice group, *r*_s_*s*(*N* = 23) < − 0.45, *p*s < 0.03.

In summary, 31% of the sequences were reproduced correctly after extended and 6% after limited practice. The number of correct responses was higher for the Spatial than the Verbal test, for BP than RP participants, and on Day 2 than on Day 1. There was no overnight consolidation difference for BP and RP participants. Participants with more spatial awareness, especially of the Extended-Practice group, executed the familiar sequences in the Retention and the Single-stimulus condition faster and were also faster with the novel sequences. Only in the Single-stimulus condition execution errors correlated with higher (spatial and verbal) awareness, especially in the Extended-Practice group.

### Discussion

As predicted, Experiment 2 showed that CI did not affect retention of the 7-key sequences (Fig. 7). The results showed the expected relatively slow R_5_ at the group level during practice and retention that confirmed that these 7-key sequences involved two successive representations for most participants (cf. Abrahamse et al., 2013; Acuna et al., 2014; Verwey, 2001; Verwey & Dronkers, 2019; Wymbs et al., 2012). Nevertheless, even with these 7-key sequences RP showed more overnight consolidation than BP (Fig. 8), but this time it cannot be attributed to improved explicit sequence knowledge on Day 2 in RP participants.

Extensive practice on Day 1 did not only improve execution of the practiced sequences in the Retention and Single-stimulus conditions on Day 2, but in both CI groups it facilitated also the production of Novel and Unstructured sequences (Fig. 9). Detailed analyses suggested that extended practice independently improved sequence representations at the motor level (indicated also by R_5_ becoming relatively fast), general sequencing skill, and the skill to react to the stimuli that had been used during practice.

Participants showed limited awareness of the three 7-key sequences, perhaps because sequences with this length are hard to distinguish. Explicit sequence knowledge seems to have contributed little to sequence execution rate, except in the slowly executed Single-stimulus condition where less aware participants also made more errors. Again, the more aware participants executed not only the familiar but also the Single-stimulus and Novel sequences faster. This corroborates the suggestion in Experiment 1 that more aware participants possess superior processing skills.

## General discussion

The contextual interference (CI) literature posits that practicing in a high CI situation promotes retention one or more days after practice while it depresses acquisition performance. We tested with the often-used discrete sequence production (DSP) task the hypothesis that high CI (during RP) would afford greater skill retention than low CI (during BP) with short sequences that are briefly practiced, and that retention would not benefit from high CI when sequences are longer or when practice is more extensive. We further examined whether the beneficial effect of high CI may be associated with differences in overnight skill consolidation, and whether high CI levels facilitate the development of reaction skill, general sequencing skill, and implicit and explicit sequence knowledge. The results are in line with the predictions.

### The CI effect

As predicted by C-SMB, the results indicated that the retention benefit of RP over BP was limited to 4-key motor sequences that had been briefly practiced, and this benefit emerged in both initiation and execution of the 4-key sequences. The CI-effect was found neither with briefly practiced 7-key sequences nor with extensively practiced 4- and 7-key sequences. These limitations of the CI effect help understand why high CI do not always improve the effects of training (Barreiros et al., 2007; Brady, 2008; Farrow & Buszard, 2017; Wulf & Shea, 2002). That the 7-key sequences of Experiment 2 did not benefit from RP, even with limited practice, supports the notion that CI is elevated during BP because the 7-key sequences involve two successively executed segments. This notion was confirmed by the observation of a slow R_5_ at the end of practice and during retention (cf. Abrahamse et al., 2013; Acuna et al., 2014; Verwey, 2001; Verwey & Dronkers, 2019; Wymbs et al., 2012). That the CI effect was not found after extended practice can be attributed to the development of sequence learning at the motor level that is not limited to short segments and that is not susceptible to the level of CI during practice.

### Underlying mechanisms

The results are in line with the notion that the benefit of RP over BP with limited practice is based on overnight consolidation and we found that this occurred with both implicit and explicit central-symbolic sequence knowledge (Kantak et al., 2010; Kim & Wright, 2020; Lin et al., 2011). In addition, improved retention seems based also on improved general sequencing and reaction skills. The improved development of reaction skill during RP is probably caused by participants relying more on the key-specific stimuli during RP because their sequences changed all the time. If so, this reaction skill is most likely limited to the actual stimuli and responses used (e.g., Logan, 1988). Interestingly, while Experiment 2 did not show the CI effect, it still showed overnight consolidation for Limited-Practice RP participants practicing 7-key sequences (Fig. 8).^4^

Extended practice appeared to foster several skills too. As expected, Experiment 2 showed that extensive practice of the 7-key sequences on Day 1 during RP and BP did not only improve performance on Day 2 in the Retention and Single-stimulus conditions, but it facilitated also the execution of Novel and Unstructured sequences. This shows that extended practice strengthened not only sequence representations but also general sequencing and reaction skills. In addition, BP participants in Experiment 1 developed with extended practice the skill to optimally prepare for initiating and executing 4-key DSP sequences before the first key-specific stimulus is displayed. This was indicated by the better performance in the Single-stimulus and Novel conditions for BP than RP participants. It cannot be attributed to just improved timing as that would not have benefited BP more than RP. This preparation skill was of course beneficial here because the tests on Day 2 involved a blocked protocol.

### Explicit sequence knowledge

The awareness task showed that most participants in both experiments did not possess full explicit sequence knowledge and the knowledge they did have was primarily spatial. Awareness was limited, especially with the three 7-key sequences. Even after extended practice less than a third of these sequences was reproduced correctly in the awareness task. In “Experiment 1”, after limited practice awareness of the 4-key sequences appeared to benefit more from overnight consolidation after RP than after BP. As the test conditions in “Experiment 1” involved a blocked regime in which participants always knew what sequence to produce, they are likely to have used this explicit knowledge for preparing and increasing performance during Retention and the Single-stimulus conditions on Day 2 (Verwey, 1996).

Both experiments confirmed earlier findings that more aware participants are faster on the practiced sequences, and that this can be observed especially after limited practice (Cleeremans & Sarrazin, 2007; Verwey, 2015; Verwey & Wright, 2014; Verwey et al., 2010). While this finding is usually taken to argue that participants use their explicit sequence knowledge for executing sequences, the present data revealed that more aware participants were also faster when executing the novel and unstructured sequences. This suggests that more aware participants possess superior processing capabilities, like a larger short-term memory capacity (Seidler et al., 2012) and/or faster information processing (Salthouse et al., 1998). So, while explicit sequence knowledge may indeed be used by more aware participants to produce familiar sequences at lower execution rates, like in the Single-stimulus condition (Verwey, 2015; Verwey & Wright, 2014; Verwey et al., 2010), at higher execution rates the more aware participants seem faster primarily because of their superior processing abilities. These superior abilities may have been the reason that they possessed more awareness of the sequences in the first place (because especially these particpants simultaneously tested hypotheses about element order, Haider & Frensch, 2009; Rünger & Frensch, 2008).

### Theoretical implications

The present findings support the proposal in the Introduction that limited capacity central-symbolic representations develop due to repeated preparation in short-term memory, which most likely also underlies the beneficial effect of mental practice (Sheahan et al., 2016; Sobierajewicz et al., 2017), while sequential motor representations result from frequent execution inducing R-R associations at the motor processing level (cf. Lindsey & Logan, 2019; Logan, 2020). Activation spreading across successive response representations is corroborated by the observation in Experiment 1 of an execution rate increase across successive responses in the 4-key sequences in the Single-stimulus condition and that the CI effect during retention increased across successive responses (Brown & Carr, 1989; MacKay, 1982; Verwey, 1994). Development of R-R associations is in line also with the finding in Experiment 2 that the typically slower concatenation response R_5_ in the 7-key sequences that was observed after 24 trials per sequence—and that we now attribute to concatenating central-symbolic representations—was no longer significantly slower than the surrounding execution responses after 504 trials per sequence. This corresponds with earlier findings in DSP experiments that after about 500 practice trials the concatenation response reaches statistical significance only in some and not in other studies, and that this slowing seems to reduce with more extensive practice in other motor sequencing studies (Acuna et al., 2014; Ramkumar et al., 2016; Wymbs et al., 2012).

These findings corroborate the idea that the segmentation of sequences is caused solely by the limited capacity of central-symbolic representations and not by sequence learning at the motor processing level (as has previously been suggested in Abrahamse et al., 2013; Verwey, 2001). The reducing reliance on central-symbolic representations when sequential motor representations develop is consistent with findings that only in early practice learning rate and segmentation pattern correlate with the capacity of visuospatial short-term memory capacity (Seidler et al., 2012), that performance becomes more effector-dependent with practice (Verwey & Wright, 2004; Verwey et al., 2009), and that neural activity shows a posterior shift in the brain during practice because other neural networks get involved (Ashby et al., 2007; Hélie et al., 2015; Verwey et al., 2019). So, we argue that the indications for concatenation with relatively short, discrete sequences can be attributed solely to central-symbolic representations that develop because of repeated preparation in short-term memory, and that with more extensive practice reliance on these central-symbolic representations reduces because sequence execution is increasingly controlled by activation of successive response at the motor level of processing (cf. Lindsey & Logan, 2019; Logan, 2020).

A core assumption of C-SMB is that processors are racing to trigger each next response in a familiar sequence. This predicts that execution rate reduces when one of the processors is no longer involved (Verwey, 2001, 2003; Verwey et al., 2015). Support for this prediction comes from Experiment 2 where the benefit of extended practice was largest for the Retention and the Single-stimulus conditions, smaller for the Novel, and smallest for the Unstructured condition. However, in “Experiment 1” the benefit on Day 2 of RP over BP in the Limited-Practice group was of a similar size for the Retention, Novel, and Unstructured conditions. We surmise that we there did not obtain evidence for the race model because execution rate of the simple 4-key sequences in “Experiment 1” had already reached its ceiling in the various test conditions, and removing a processor from the race did reduce execution rate.

## Additional issues

A limitation of the present study is the use of a blocked testing protocol. We used it to demonstrate that even in that situation RP yields better retention than BP, but using a BP test protocol does imply that the present study focused on execution skill and not on the skill to select and prepare sequences.

Another limitation of the present study is the use of a 24-h retention interval. A few studies showed that the retention benefit of RP continued to increase across successive days (Kim & Wright, 2020; Walker et al., 2003a, 2003b; Walker et al., 2003a, 2003b). This was especially due to RP resisting the typical forgetting over days that is observed after BP. Future research might therefore assess retention after RP and BP after more than a single day.

The results obtained with the 7-key sequences in the Single-stimulus condition suggest that after limited practice participants prepared the first four responses of the 7-key sequences at the start of a block in short-term memory, probably using especially explicit sequence knowledge (cf. Cowan, 2000), and only then executed these responses (Verwey, 1996). The ensuing three responses were then produced one by one in a slow manner, probably because they were using explicit sequence knowledge. As suggested by the high error rate of RP participants in early practice of the 7-key sequences, this strategy may have been used also in early practice on Day 1 (Fig. 6, left frame), which, therefore, eventually prompted the concatenation point at the fifth sequence position. This interpretation explains why the segments observed so often with 6- and 7-key DSP sequences after moderate practice fit the capacity of short-term memory, and that there is no need to assume a motor buffer that coincidentally has the same limited capacity as short-term memory (Abrahamse et al., 2013; Verwey & Eikelboom, 2003).

## Conclusions

The results of Experiments 1 and 2 corroborated the hypotheses that were based on the C-SMB, and they led to the following conclusions. (1) The CI-effect is limited to short motor sequences that are briefly practiced and does not occur with longer sequences and sequences that have been practiced extensively. This is accounted for by the notion that central-symbolic representations develop due to frequent preparation in short-term memory whereas the R-R associations underlying learning at the motor level gradually develop when motor sequences are frequently executed. The benefit of RP over BP seems associated with overnight consolidation. (2) Increased CI by RP of short sequences fosters implicit and explicit sequence knowledge (due to overnight consolidation), general sequencing skill, and reaction skill. (3) Longer sequences, like the 7-key sequences, show no learning benefit of limited practice RP because concatenation of successive central-symbolic representations already increases CI during BP. Still, RP did induce overnight consolidation for these longer sequences too. (4) Extended practice in both the BP and RP regimes boosts general sequencing and reaction skills while extended BP promotes the ability to prepare short sequences. (5) The race assumption of C-SMB is corroborated by the finding that sequence execution rate increased when in “Experiment 2” more cognitive processors were involved. Not finding this in “Experiment 1” can be attributed to execution rate of short sequences reaching its maximum even when not all systems are involved. (6) Explicit sequence knowledge is used only for execution when execution rate is low or there is ample time to prepare like in a blocked regime. Previous findings that moderately practiced sequences are executed faster by more aware participants faster can be attributed to superior processing abilities of these participants rather than to application of explicit sequence knowledge. These superior abilities may in fact have been responsible for their higher awareness in the first place.

## Footnotes

1 Verbal knowledge may develop after executing keying sequences in the DSP task, but verbal coding may also precede the execution of these motor sequences (De Kleine & Verwey, 2009; Verwey, Lammens, & van Honk, 2002), like when learning to type in one’s ATM pin number on a keyboard with an unfamiliar layout (Krakauer, Hadjiosif, Xu, Wong, & Haith, 2019; Verwey, 2015).

2 Central-symbolic and sequential motor representations may well be based on the same associative chaining mechanism. This consists of associations developing between low-level representations that are repeatedly active at the same time (Abrahamse, Jiménez, Verwey, & Clegg, 2010; Lindsey & Logan, 2019; MacKay, 1982, 1990). There is now extensive neural evidence that associative learning is a general learning mechanism that occurs in sensory and motor neural systems (Beukema & Verstynen, 2018; Meyer & Olson, 2011; Mushiake, Inase, & Tanji, 1991) and in perception, memory, and action (Logan, 2020).

3 Balancing of the 4 available sequences across participants assured that each sequence was equally often represented in the first and last parts of practice for each of the 4 groups. Sequences with an error were excluded from this set of 12.

4 The general awareness improvement on Day 2 for RP and BP participants cannot be attributed to consolidation because it may have also resulted from experience with the awareness task on Day 1.
